# Injuries of the Eye

**Published:** 1896-11-21

**Authors:** E. Treacher Collins


### INJURIES OF THE EYE.

By E. Treacher Collins, F.R.C.S.

Abstract of a Post-graduate Lecture delivered at the Royal London Ophthalmic Hospital, Moorfields, on November 2nd, 1896.

Injuries of the eye may be conveniently divided for practical purposes into those in which there is a perforation of the globe and those in which there is not. Under the latter are included foreign bodies in the cornea or beneath the lid, abrasions of the cornea, wounds of the conjunctiva, burns of the eye, concussion injuries, and subconjunctival ruptures.

To remove a foreign body from the cornea, after the application of cocaine to the eye, the patient should be seated opposite a good light. The surgeon should stand behind and separate the lids by inserting the two first fingers of his left hand into the palpebral fissure and drawing them apart. At the same time he should, by pressure backwards of the fingers, fix the globe and prevent its rolling about, while with a blunt-pointed spud he partly levers and partly scrapes the foreign body away.

In cases of abrasion of the cornea it is not advisable to prescribe cocaine; the relief of pain which it affords lasts at the outside only about three-quarters of an hour, and it tends to soften the epithelium and delay healing, so that very frequently under such treatment the wound will become infected and a spreading ulcer form. In such cases tying the eye up with a compress and the application of atropine affords a more permanent relief of pain. Slight abrasions of the cornea are often very difficult to detect, but are readily shown up by the application of a drop of a two per cent. solution of fluorescine, when the denuded area will stain a bright green.

In a recent case of burn of the cornea and conjunctiva, or destruction with lime, strong acids, or alkalies, any of the damaging substance should, if still present, at once be removed, every particle of lime should be sought for and wiped away, and any acid or alkali neutralised by a weak lotion of the opposite reaction. If the corneal epithelium alone is damaged it will quickly reform and no permanent injury result, but usually the deeper tissues are also involved and a considerable slough forms. In such cases a very guarded prognosis should be given when the patient is first seen, as the condition tends to become more severe than it appears at first. In cases where there is a large area distroyed it is not well to tie up the eye, as plastic exudation is thrown out, and the lid becomes united to the globe (symblepharon). The patient should be instructed to draw the lid away from the eye several times a day so as to break down such adhesions.

As the result of concussion of the eye from a blow on it by a blunt object several lesions may occur, such as separation of the iris from the ciliary body (iridodialysis); rupture of the sphincter muscle; rupture of the capsule of the lens and the formation of cataract; rupture of the suspensory ligament and dislocation of the lens, and rupture of the choroid. In concussion injuries there is often at first much intraocular hæmorrhage, and the exact extent and nature of the injury cannot be diagnosed until it has absorbed. The first treatment in such cases is the application of atropine and a cold compress.

Ruptures of the sclerotic are usually situated 2 or 3 mm. from the sclero-corneal margin and concentric with it, either upwards or upwards and inwards, *i.e.*, in the opposite position to that in which the blow is inflicted. The conjunctiva over the rupture sometimes remains intact; then, if there is any escape of the contents of the globe, such as the lens or iris, they lie beneath it. In a case of subconjunctival dislocation of the lens, the lens should, if possible, be left until the scleral wound has healed, and then removed, otherwise the wound, which is at first simple, will be made compound. In many cases of rupture of the globe the damage to its contents is so great that it is best to at once excise the eye.

Whenever there has been an opening made into the globe the possibility of the occurrence of sympathetic ophthalmia has been established; the probability of its coming on depends on the position of the wound, the character and condition of the wounding substance, and the amount of reaction that follows. Operations on the eye which are planned to avoid the ciliary body (the dangerous region), and which are performed with aseptic instruments, are rarely followed by sympathetic mischief, though occasionally it does occur after operations for cataract.

Simple wounds of the cornea generally heal well and quickly. In a wound of the cornea with a prolapse of iris, it is important to draw out and cut off the prolapse as early as possible, before it has had time to form adhesion to the sides of the wound. If the lens is wounded, it is liable to swell up and give rise to increase of tension, pain, and irritation, in which case it requires to be let out. Eyes with wounds of the ciliary body and lens should be at once excised. The amount of useful sight which the patient is likely to retain in such an eye is not sufficient to make it worth his while running the great risk of sympathetic ophthalmia which a wound in that situation entails. Wounds of the ciliary body, in which the lens is not involved and remains clear, are exceedingly difficult cases in which to decide what course is best to pursue. It is generally well to watch the case for a few days, and, if much reaction sets in, to at once advise excision.

If a foreign body becomes lodged in the eye, an endeavour must first be made to determine its nature from the history of the accident. If it is a piece of steel, an attempt should be made to remove it with the electro-magnet. This is most likely to prove successful if it is situated in the anterior chamber or lens. If a foreign body cannot be removed from an eye, the eye generally has to be excised. In a few exceptional cases it may be left, such as those in which the foreign body can be seen with the ophthalmoscope in the vitreous behind the ciliary region, and where the eye remains free from irritation. A shot will sometimes pass right through an eye and become lodged in the orbit; in these cases, occasionally, the aperture of entrance and exit of the shot can be seen with the ophthalmoscope.

